# Reduced Serum Levels of Bone Formation Marker P1NP in Psoriasis

**DOI:** 10.3389/fmed.2021.730164

**Published:** 2021-10-01

**Authors:** Julia Mentzel, Tabea Kynast, Johannes Kohlmann, Holger Kirsten, Matthias Blüher, Jan C. Simon, Manfred Kunz, Anja Saalbach

**Affiliations:** ^1^Department of Dermatology, Venereology and Allergology, University Medical Center Leipzig, Leipzig, Germany; ^2^Institute for Medical Informatics, Statistics, Epidemiology, Medical Faculty of Leipzig University, Leipzig, Germany; ^3^Medical Department III, Endocrinology, Nephrology, Rheumatology, University Medical Center Leipzig, Leipzig, Germany

**Keywords:** psoriasis, chronic inflammatory diseases, bone metabolism, osteoporosis, skin

## Abstract

Psoriasis is a chronic inflammatory disease of the skin and joints. More recent data emphasize an association with dysregulated glucose and fatty acid metabolism, obesity, elevated blood pressure and cardiac disease, summarized as metabolic syndrome. TNF-α and IL-17, central players in the pathogenesis of psoriasis, are known to impair bone formation. Therefore, the relation between psoriasis and bone metabolism parameters was investigated. Two serum markers of either bone formation—N-terminal propeptide of type I procollagen (P1NP) or bone resorption—C-terminal telopeptide of type I collagen (CTX-I)—were analyzed in a cohort of patients with psoriasis vulgaris. In patients with psoriasis, P1NP serum levels were reduced compared to gender-, age-, and body mass index-matched healthy controls. CTX-I levels were indistinguishable between patients with psoriasis and controls. Consistently, induction of psoriasis-like skin inflammation in mice decreases bone volume and activity of osteoblasts. Moreover, efficient anti-psoriatic treatment improved psoriasis severity, but did not reverse decreased P1NP level suggesting that independent of efficient skin treatment psoriasis did affect bone metabolism and might favor the development of osteoporosis. Taken together, evidence is provided that bone metabolism might be affected by psoriatic inflammation, which may have consequences for future patient counseling and disease monitoring.

## Introduction

Bone is a dynamic tissue that is constantly being remodeled in a lifelong process with about 10% of bone material being renewed each year (1). It involves the concerted action of osteocytes, osteoclasts, and osteoblasts. In normal bone remodeling, a proper balance between bone resorption and formation is controlled by coordinated signaling mechanisms (1).

An imbalance in bone resorption and bone formation may occur under certain pathological conditions, which lead to abnormal bone remodeling and the development of bone disorders. Osteoporosis is a common disorder of bone remodeling characterized by a reduction in bone mineral density (BMD) with impaired bone microarchitecture, reduced strength and increased fracture risk. Primary osteoporosis is caused by postmenopausal estrogen deficiency or by age-related changes. In contrast, secondary osteoporosis is related to secondary complications such as vitamin D or calcium deficiency, changes in physical activity, or therapeutic interventions like long-term glucocorticoid treatment (1). Inflammation has been identified as a potential risk factor for osteoporosis. Proinflammatory cytokines such as TNF-α, IL-6, and IL-1β are important mediators of bone resorption supporting osteoclast differentiation, activation, and survival, enhancing RANKL expression and inhibiting osteoblast survival resulting in increased bone resorption (2). Recently, a promotion of bone loss by stimulating osteoclast formation and inhibiting osteoblast differentiation by IL-17 has been reported (3, 4). Psoriasis is a chronic inflammatory skin disease associated with several comorbidities including metabolic syndrome, obesity, hypertension and dyslipidaemia, diabetes mellitus, psoriatic arthritis, Crohn's disease, uveitis and psychiatric and psychological disorders (5, 6). Interestingly, TNF-α and IL-17, cytokines known to impair bone formation, are central players in the pathogenesis of psoriasis (7–9). However, epidemiological studies revealed contradictory results regarding osteoporosis risk in psoriatic patients. Two large-scale population studies supported a positive association. Analysis of the longitudinal health insurance database in Taiwan with 17,507 osteoporosis patients and 52,521 controls indicated that osteoporosis was significantly associated with a previous diagnosis of psoriasis in both sexes (10). In a large cross-sectional study in the US, an association of psoriasis and psoriatic arthritis with osteopenia, osteoporosis, ankylosing spondylitis has been observed (11). Interestingly, psoriatic patients with osteopenia/osteoporosis showed significant longer average duration of psoriatic disease compared to patients with healthy bones indicating the necessity of an early diagnostic evaluation of bone metabolism in patients with psoriasis (12). In contrast, in the HUNT3 study including 48,194 participants, no association between psoriasis and bone fracture risk, reduced BMD, *T*-score or higher prevalence of osteoporosis has been found among patients with psoriasis (13). The failure of an association of psoriasis and osteoporosis was confirmed by several smaller studies (14–17). Thus, the relationship between psoriasis and osteoporosis is still an open question. Here, we examined serum levels of P1NP and CTX-I as serum markers of bone formation and resorption, respectively, in the serum of patients with psoriasis vulgaris and age-, gender- and weight-matched healthy controls. Our data showed that the bone formation marker, P1NP, was significantly decreased in serum of patients with psoriasis vulgaris while the bone resorption marker, CTX-I, was not changed. Moreover, induction of psoriasis-like skin inflammation in mice impaired bone metabolism. These data suggest that psoriasis might be accompanied by alterations in bone formation.

## Materials and Methods

### Patients

#### Cohort 1

Serum was collected from 64 patients with psoriasis vulgaris and 49 healthy controls which have been group-matched for age, sex and body mass index (BMI) (Table 1). Patients with psoriasis vulgaris and healthy controls without diabetes were included. To exclude any effects of post-menopausal estrogen deficiency and aging on bone metabolism, we only used pre-menopausal women with an age below 55 and men below 60. Psoriasis had to be diagnosed for at least 6 month. Ten patients showed nail involvement. None of the patients showed clinical signs of joint involvement. Patient treatment included topical treatment, UV-light, and systemic pharmacological treatment (Table 1).

**Table 1:** Patient characteristics cohort 1.

	**Control**	**Psoriasis vulgaris**		***p*** **-value**
**Number**	49	64		
**Females**	**25 (51%)**	**25 (39%)**		**0.25**
**Males**	**24 (49%)**	**39 (61%)**		**0.25**
**Age, mean (years)**	43 ± 10.12	44 ± 11.51		**0.46**
**Females**	38.0 ± 6.7	42 ± 11.8		0.72
**Males**	31.25 ± 12.4	46 ± 11		0.75
**Body mass index**	27.2 ± 4.32	29.7 ± 9.1		0.13
**Females**	24.3 ± 4,37	26.16 ± 6.5		0.85
**Males**	28.55 ± 4.17	29.2 ± 9.8		0.053
**Diabetes**	**0 (0%)**	**0 (0%)**		1.00
**Systemic therapy**		**24 (38%)**		
Fumaric acid		5		
Acictretin/retinoids		1		
PDE_4_-inhibitor		1		
Methotrexate		4		
Glucocorticoids		1		
Anti-TNFα		3		
Anti-IL-23		1		
Anti-IL12/23		1		
Anti-IL-17		7		
**PASI, mean**		**Female**	**Male**	
		12.5 ± 7.17	17.65 ± 12.8	0.04
		**BMI < 25**	**BMI > 25**	
		13.6 ± 7.7	16.95 ± 12.74	0.11

#### Cohort 2

Serum was collected from 10 patients with psoriasis vulgaris before and 3 month after successful anti-psoriatic treatment with biologics (Table 2).

**Table 2:** Patient characteristics cohort 2 and 3.

**Cohort 2**		
Number	10	
Females	2 (20%)	
Males	8 (80%)	
Age	44.9 ± 10.18	
BMI	30.74 ± 9.2	
Treatment	Ixekizumab Adalimumab Tildrakizumab Brodalumab	
	Before	After
PASI	17.8 ± 5.7	3.8 ± 2.1
**Cohort 3**		
Number	13	
Females	2 (15%)	
Males	11 (85%)	
Age	56.7 ± 9.8	
BMI	29.2± 5.3	
Duration of treatment	2.6 ± 3.0 years	
Treatment	Ustekinumab Guselkumab, Infliximab Ixekizumab, Tildrakizumab, Adalimumab, Etanercept, Risankizumab, and Brodalumab	
	Before	After
PASI	12.8 ± 3.7	3.1 ± 3.0

#### Cohort 3

Serum was collected from 16 patients with psoriasis vulgaris after at least 6 month of systemic treatment (Table 2). Psoriasis Area and Severity Index (PASI), body weight, and height were determined.

The study was approved by the local ethics committee (# ethics vote: 332/13-ff, 161/18-ek). All patients gave their written consent before taking part in the study.

### ELISA

Human P1NP and CTX-I serum levels were detected by ELISA according to the manufacture's specifications (Abexxa, Cambridge, UK). Serum concentrations of mouse TNFα was detected using immunoassay kit (Thermo Fisher Scientific, Worldham, MA, USA) according to the manufacturer's protocol.

### Mouse Studies

A psoriasiform skin inflammation was induced by topical application of 60 mg imiquimod (IMQ, Aldara, Meda GmbH, Wiesbaden, Germany) on the shaved back of male C57/BL6 mice every 3 days over 3 weeks. All animal experiments were performed according to institutional and state guidelines. The Committee on Animal Welfare of Saxony (Germany) approved animal protocols used in this study (TVV65/13). During sacrifice, blood was drawn by heart punctuation, centrifuged and serum was frozen at −80°C. Bones were fixed in 4% paraformaldehyde (PFA, Carl Roth) for μCT analysis or frozen for RNA isolation.

### Assessment of Bone Mass

PFA fixed bones were dehydrated in 80% ethanol. Femora were analyzed by μCT vivaCT40 (isotropic voxel size of 10.5 μm; 70 kVp, 114 μA, 200 ms integration time, ScancoMedical), as previously described (18). The analysis of trabecular and cortical bone volume per total volume (BV/TV) was performed using established analysis protocols (18).

### RNA Isolation and Quantitative Real Time PCR

RNA from frozen bone samples (ulnae) was isolated using Trifast (Peqlab, Erlangen, Germany) according to the manufacturer's instructions. One microgram of total RNA was used for first strand cDNA synthesis with M-MLV reverse transcriptase (Promega, Mannheim, Germany) according to the manufacturer's protocol. Real-time qPCR was performed with *GoTaq*^®^ *qPCR Master* (Promega) according to the manufacturer′s instructions on RotorGeneQ (Qiagen, Hilden, Germany). Runx2 forward primer: CTCCAAGACCCTAAGAAACCG; Reverse primer: TCTCTCAGATACCATGGGTGC; TNALP forward primer: TGTGGTTACTGCTGATCATTC; Reverse primer: TTGTGAGCGTAATCTACCATGG; Rs36: forward primer: GGACCCGAGAAGACCTCCTT; Reverse primer: GCACATCACTCAGAATTTCAATGG. Quantitative gene expression was calculated from the standard curve of cloned cDNA and normalized to the unregulated reference genes *Rs36*.

### Statistical Analysis

Statistical analysis for two-group comparisons regarding normally distributed metrical data was performed using two-tailed Student's *t*-test (not assuming equal variance between cases and controls, i.e., applying the Welch approximation to the estimation of degrees of freedom). Normality was tested by D'Agostino and Pearson Normality test or Shapiro–Wilk-Test (*n* ≤ 4). Where normality was absent, Mann–Whitney test was used. For comparison of categorical data, Fisher's exact test was used.

For statistical comparison of three groups, we used Welch's ANOVA Test not assuming equally variance across groups. As *post-hoc* test in case of significance of the ANOVA, we used Dunnet's T3 multiple comparison test. Calculations were done using GraphPad Prism version 7.02. *P*-values of 0.05 or smaller were considered statistically significant.

## Results

### Psoriatic Patients Display Decreased Levels of Bone Formation Marker P1NP

**Figure 1:**
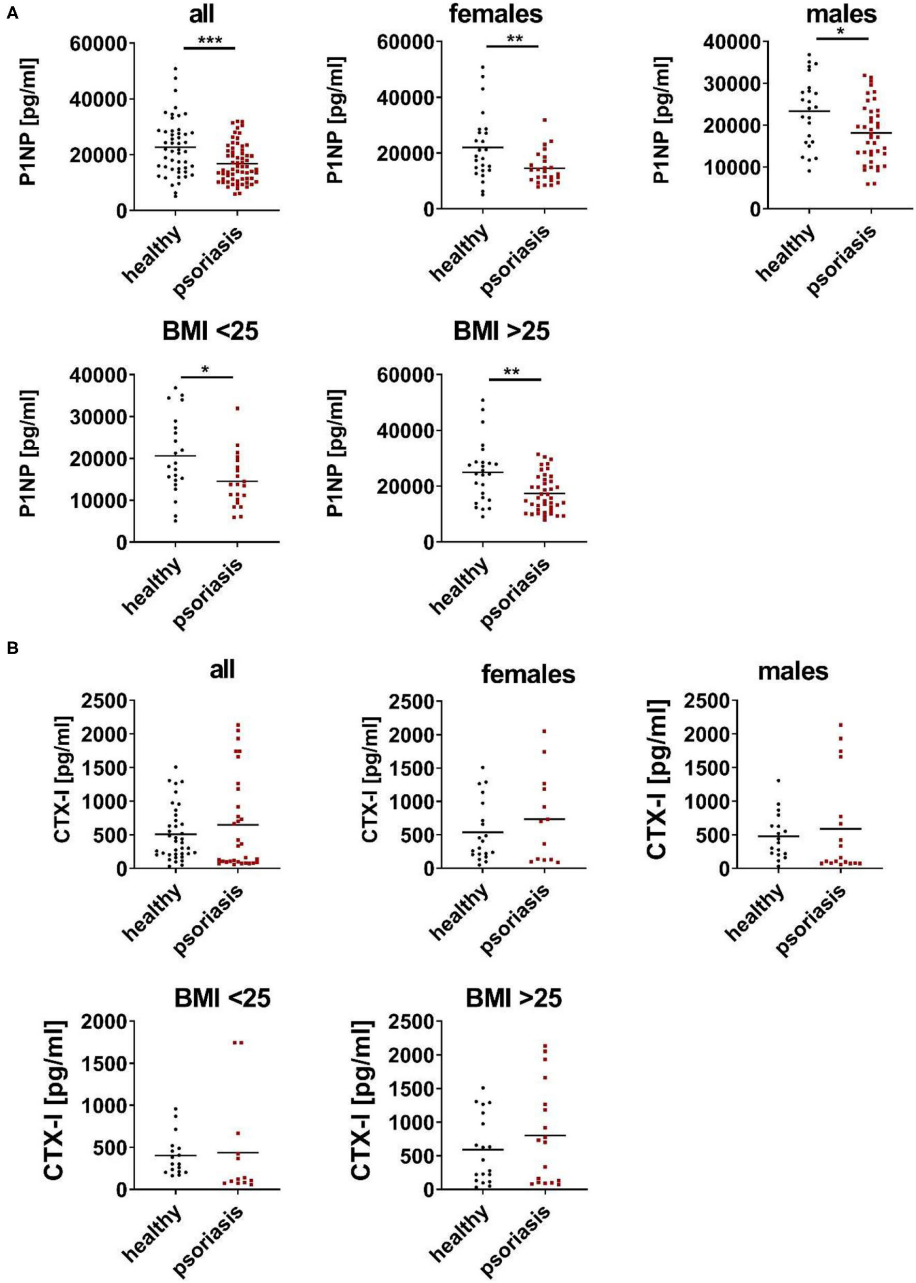
Serum P1NP is reduced in psoriatic patients. **(A,B)** N-terminal propeptide of type I procollagen (P1NP) **(A)** and C-terminal telopeptide of type I collagen (CTX-I) **(B)**—were analyzed in serum of psoriatic patients (red) and age, gender, and body mass index (BMI) matched healthy controls (black) in cohort 1. Each point represents one patient. *p < 0.05; **p < 0.01; ***p < 0.001.

To understand the relation of psoriasis and bone metabolism we analyzed serum bone parameters of individuals with psoriasis vulgaris and controls. Since P1NP and CTX-I are preferred for clinical use as a marker of bone formation and resorption, respectively, we measured both in serum of patients with psoriasis vulgaris and age/gender matched healthy subjects (17–19). As shown in Figures 1A,B, psoriatic patients displayed significant lower levels of P1NP while no significant differences were observed for CTX-I levels. Separate analysis of men and women confirmed the reduction of P1NP in both sexes (Figures 1A,B). Again, no significant differences were observed for levels of CTX-I. Since there is accumulating evidence that increased abdominal fat might be a risk factor for decreased BMD and osteoporosis in women and men (20), we analyzed P1NP and CTX-I in relation to body mass index (BMI). P1NP was decreased in both lean (BMI <25) and obese (BMI > 25) psoriatic patients compared to healthy BMI-matched controls while for CTX-I, no significant differences were observed (Figures 1A,B).

**Figure 2:**
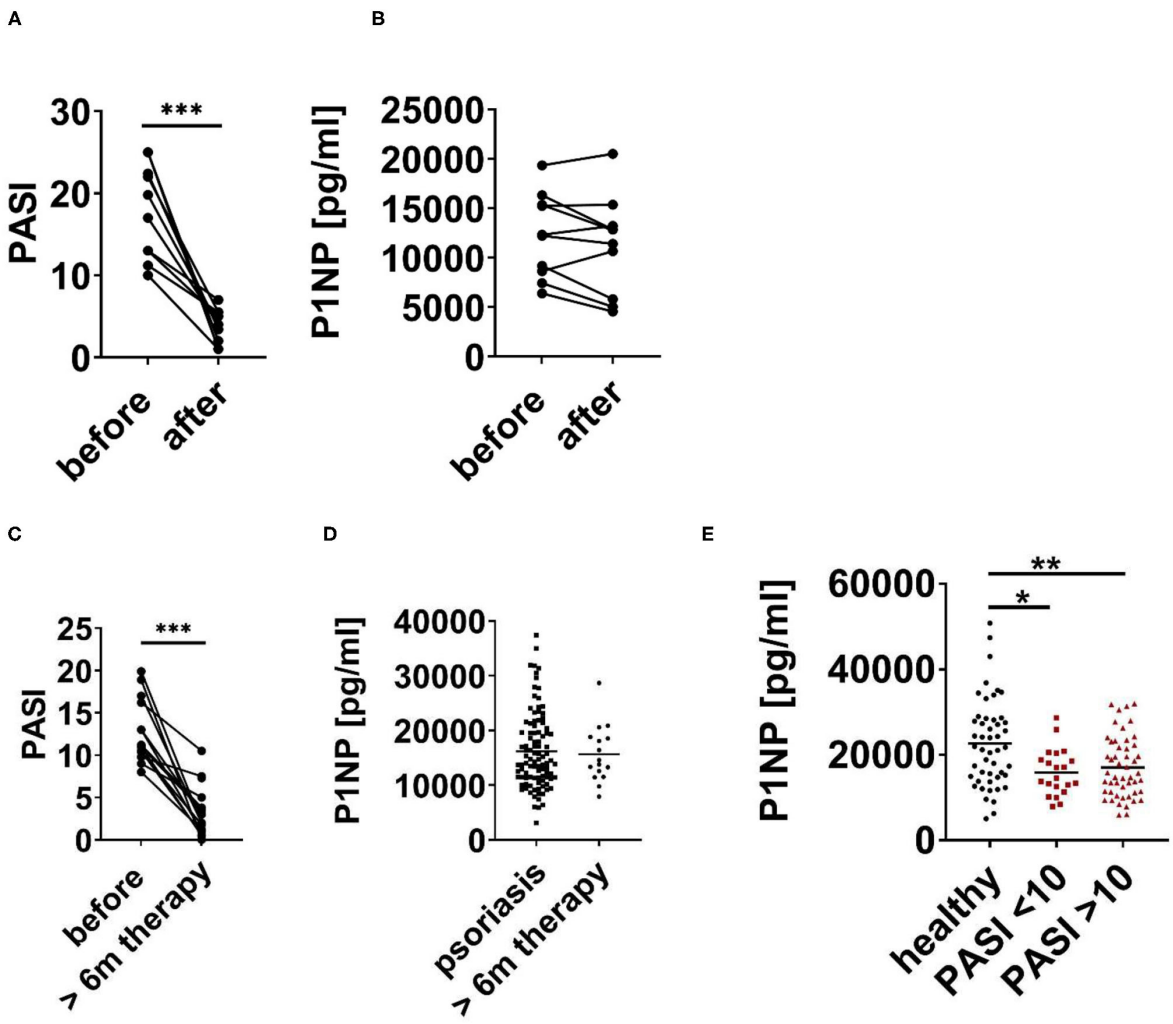
P1NP before and after anti-psoriatic treatment. **(A,B)** Psoriatic patients before and after 3 month of anti-psoriatic treatment with biologics. **(A)** PASI and **(B)** Serum N-terminal propeptide of type I procollagen (P1NP). **(C)** PASI of psoriatic patients before and after at least 6 months (6 m) of treatment. **(D)** P1NP in serum of psoriatic patients before (cohort 1–3) and after at least 6 months of anti-psoriatic treatment with biologics (cohort 3). **(E)** P1NP in healthy subjects (cohort 1), in all psoriatic patients with PASI < 10 and PASI > 10 (cohort 1–3). Each point represents one patient. *p < 0.05; **p < 0.01.

Next, we checked the impact of efficient anti-psoriatic treatment on serum bone turnover markers. Serum of patients with psoriasis before and after 3 month of systemic treatment of psoriasis with biologics was analyzed (Table 2). As shown in Figure 2A, anti-psoriatic treatment efficiently reduced psoriasis activity in the skin represented by clear, statistically significant reduction of PASI. However, this successful treatment of skin phenotype was not associated with a significant change of the bone formation marker P1NP (Figure 2B). To exclude that 3 month of treatment was not sufficient to observe changes in bone metabolism we analyzed serum of patients with psoriasis vulgaris after at least 6 months of successful anti-psoriatic treatment (Table 2). PASI reduction proved the successful treatment as we again observed a statistically significant difference. Similarly, we did not observed any significant change of P1NP (Figures 2C,D). In addition, P1NP concentration did not differ significantly between patients with mild to moderate (PASI < 10) and severe (PASI > 10) disease, however, for both groups the difference to the controls was significant (Figure 2E).

In summary, our data show that psoriasis is associated with decreased levels of bone formation marker P1NP independent of disease severity and successful anti-psoriatic treatment of the skin phenotype.

### Psoriasis-Like Skin Inflammation in Mice Reduces Bone Volume

To provide supporting evidence for the relationship of psoriatic skin inflammation and bone metabolism and due to the limitation in patients we analyzed the bone in a psoriasis-like inflammation model in the mouse. Repetitive application of imiquimod on shaved back induces a psoriasis-like skin inflammation phenotype in mice (19). Consistently, we found elevated levels of serum TNFα, one important pathogenic mediator in psoriasis (Supplementary Figure 1A). Importantly, the bone volume per total volume (BV/TV) of the trabecular and cortical bone compartments detected by μCT was reduced in mice with psoriasis-like skin inflammation compared to untreated healthy mice (Supplementary Figure 1B). Moreover, expression of alkaline phosphatase (TNALP), a major regulator of bone mineralization, and runx2, the master transcription factor for the osteogenic differentiation, were decreased in the bone of mice with psoriasis-like skin inflammation (Supplementary Figure 1C). These data underline the impact of psoriasis-like inflammation on bone formation.

## Discussion

Several epidemiological studies show an association of psoriasis and decreased BMD and increased fracture risk suggesting that patients with psoriasis have a higher risk to develop osteoporosis. However, actually screening of bone is not included in the monitoring of psoriasis patients. Measurement of BMD is the gold standard for evaluation of bone quality, but it is associated with radiation exposure. Therefore, we need additional strategies to investigate the bone metabolism in psoriasis. Bone turnover markers are important tools to screen bone metabolism. These biomarkers are produced during the bone remodeling process including markers of bone formation, resorption and regulators of bone metabolism (20, 21). Bone formation biomarkers are P1NP, total alkaline phosphatase, bone-specific alkaline phosphatase or osteocalcin. P1NP is a peptide derived from posttranslational cleavage of type I procollagen molecules by proteases during collagen deposition by osteoblasts. Bone resorption biomarkers are CTX-I, amino-terminal crosslinked telopeptide of type 1 collagen and cathepsin K. They are generated during the bone resorption phase of bone remodeling.

Among biomarkers of bone metabolism, CTX-1 and P1NP are commonly regarded as reference biomarkers for the measurement of bone resorption and formation (22). Moreover, recent recommendations by the Bone Marker Standards Working Group propose to standardize research and include a specific marker of bone resorption (CTX) and bone formation (P1NP) in all future studies (23). Epidemiological studies confirmed a strong association of bone turnover markers with fracture risk reduction during osteoporosis treatment (20, 21). Thus, postmenopausal women with low BMD display lower levels of P1NP than those with normal BMD (24). Recently, it was shown that P1NP has a significant negative correlation with BMD and quality of life of osteoporotic patients indicating that serum bone turnover markers like P1NP are an alternative to the conventional measurement of BMD (25). In another study P1NP levels differ significantly between healthy, osteopenic, and osteoporotic patients (26). Therefore, serum markers of bone metabolism are used to support management decisions for osteoporosis and for monitoring of anabolic and anti-resorbtive osteoporosis treatment (27). Taken together, there are various studies showing that P1NP and CTX-I are an alternative to monitor changes in the bone when DEXA scan is not applicable.

Based on these data we analyzed P1NP and CTX-I in serum of patients with psoriasis vulgaris and age, gender and BMI matched healthy controls. In the present study, we show a decrease of the bone formation marker P1NP in psoriatic patients independent of sex, age, and BMI, while CTX-I as bone resorption marker was unaffected. Since several studies showed that that P1NP and CTX-I reflect bone metabolism our data suggest that systemic inflammation in psoriasis does interfere with bone formation. A relationship between inflammation and bone disease has been established in a variety of inflammatory settings such as rheumatoid arthritis, spondylarthropathies, periodontal diseases, inflammatory bowel disease, coeliac disease, chronic lung inflammation including asthma, chronic obstructive pulmonary disease or alveolitis, and renal diseases (28). Osteoporosis may be explained by different mechanisms including direct effects of inflammation on BMD, the immobility of these patients due to inflammatory symptoms and/or joint destruction, an unfavorable nutritional status, and long-term use of glucocorticoids. The impact of inflammation itself on bone became clearer in a recent study of a cohort of recent-onset rheumatoid arthritis patients. In this population, accelerated bone loss was clearly associated with parameters of inflammatory activity (29). Epidemiological studies indicated that even a small rise in the level of systemic inflammation can induce bone loss and may be an independent risk factor for fractures (29). Schett (30) demonstrated that hs-CRP levels are a strong risk predictor of non-traumatic fractures in the general population.

These observations are supported by data from animal studies showing systemic bone loss in experimental models of arthritis and colitis (9, 31). Consistently, we found decreased bone volume, reduced expression of the bone formation markers TNALP and runx2 in mice with psoriasis-like skin inflammation. These data are underlined by a study of Uluckan et al. (4) showing bone loss in IL17-driven mouse models of psoriasis-like skin inflammation. In contrast to our model, the pathogenic phenotype has been established in these mice since birth. In our model only 3 weeks of skin inflammation is sufficient to impair bone showing the fast and strong effect of inflammation on bone metabolism.

These data are supported by the observation that stimulation of skin organ cultures with TNF-α, IL-17, osteopontin, or IL-33, all of which are elevated in psoriasis, resulted in the induction of pro-osteoclastogenic factors, inhibition of anti-osteoclastogenic factors and support the differentiation of monocytes into osteoclast precursors (8). In addition, in patients with rheumatoid arthritis, where IL-17 also plays a crucial pathogenic role, an enhanced risk of osteoporosis has been observed (32–35).

Our data underline that chronic inflammation in psoriasis alters bone turnover and thus psoriatic patients might have a higher risk for the development of osteoporosis. Reduced serum P1NP levels in parallel to reduced bone volume, less bone trabeculae in psoriatic patients compared to healthy controls (4) support that reduced P1NP found in our study might have clinical relevance. In addition, none of the subjects in our study reported signs of osteoporosis such as reduction in height or non-traumatic fractures. Thus, reduction of serum levels of P1NP was detectable in psoriatic patients before any clinical signs of bone alteration. One limitation of the study is that we do not have information about the physical activity of the patients and controls. Moreover, we did not observe differences in P1NP in patients with mild to moderate and severe disease activity suggesting that long term mild inflammation is sufficient to impair bone metabolism. Moreover, successful anti-psoriatic treatment of the skin phenotype did not reverse decreased P1NP level within 6 months. We have to consider that psoriatic patients were treated with different systemic therapies including different biologicals, fumaric acid, retinoids, or methotrexate. We cannot exclude that specific treatment options might improve bone formation. Taken together, our data suggest that psoriasis did affect bone metabolism and might favor the development of osteoporosis. Thus, additional bone screening of these patients may be necessary.

Recently, Guo et al. (36) addressed a similar question – the relationship of bone metabolism and diabetes. It is well-recognized that diabetes or impaired glucose metabolism could affect bone health, contributing to decreased bone formation, increased bone marrow adiposity and increased risk of fracture (37–39). Similar to our study they found that lower P1NP levels were associated with a higher prevalence of insulin resistance. HOMA-IR was negatively related to P1NP. They conclude that detection of bone turnover markers in diabetes or hyperglycemia patients helps to predict the risk of osteoporosis and fracture, relieve patients' pain and reduce the expenses of long-term cure (36).

In summary, the data presented herein, together with epidemiological studies, studies in animal models and cell culture suggest that psoriatic inflammation alters bone metabolism and might increase the risk of osteoporosis. Thus, early screening of bone quality might be helpful to prevent psoriasis-associated bone alterations.

## Data Availability Statement

The raw data supporting the conclusions of this article will be made available by the authors, without undue reservation.

## Ethics Statement

The studies involving human participants were reviewed and approved by the local Ethics Committee of the Medical Faculty of the University Leipzig. The patients/participants provided their written informed consent to participate in this study. The animal study was reviewed and approved by Committee on Animal Welfare of Saxony (Germany).

## Author Contributions

AS and MK designed the study, analyzed, and interpreted data, and wrote the manuscript. TK, JK, MK, and MB performed patient characterization and collected samples. TK and AS performed the experiments. HK performed the statistical analysis. All authors discussed the data and study design, read, and edited the manuscript.

## Funding

This work was supported by PsoNet Leipzig/Westsachsen and Hautnetz.

## Conflict of Interest

The authors declare that the research was conducted in the absence of any commercial or financial relationships that could be construed as a potential conflict of interest.

## Publisher's Note

All claims expressed in this article are solely those of the authors and do not necessarily represent those of their affiliated organizations, or those of the publisher, the editors and the reviewers. Any product that may be evaluated in this article, or claim that may be made by its manufacturer, is not guaranteed or endorsed by the publisher.

## Supplementary Material

Supplementary Figure 1 Psoriasis-like skin inflammation in mice decreases bone volume and osteoblast activity. Psoriasiform skin inflammation was induced by repetitive topical application of imiquimod (IMQ) on the shaved back of male C57/BL6 mice. Untreated mice were used as control (ctr). **(A)** Detection of TNFα in serum by ELISA. **(B)** Detection of bone volume to total volume (BV/TV) in the trabecular and cortical compartment of femora by μCT. **(C)** Quantitative PCR analysis of alkaline phosphatase (TNALP) and runx2. Each point represents one mouse. **p* < 0.05; ***p* < 0.01.
