# Supplementary figures and images for: Reduced Serum Levels of Bone Formation Marker P1NP in Psoriasis
Source: Front Med (Lausanne). 2021 Oct 1;8:730164. doi: 10.3389/fmed.2021.730164 (PMC8517119; doi:10.3389/fmed.2021.730164)

**
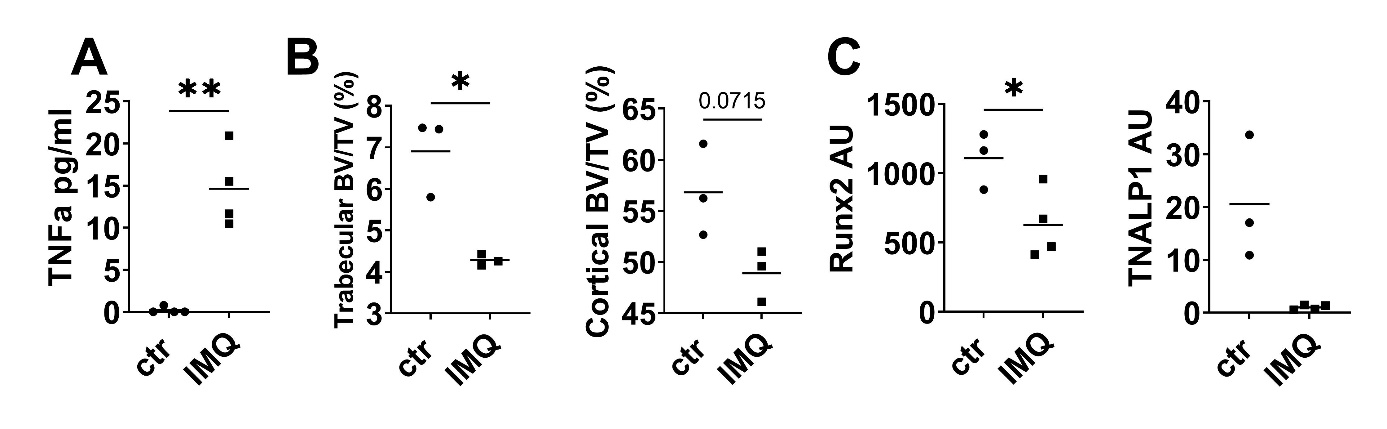
**

Supplement: Supplementary Figure 1 — Psoriasis-like skin inflammation in mice decreases bone volume and osteoblast activity. Psoriasiform skin inflammation was induced by repetitive topical application of imiquimod (IMQ) on the shaved back of male C57/BL6 mice. Untreated mice were used as control (ctr). (A) Detection of TNFα in serum by ELISA. (B) Detection of bone volume to total volume (BV/TV) in the trabecular and cortical compartment of femora by μCT. (C) Quantitative PCR analysis of alkaline phosphatase (TNALP) and runx2. Each point represents one mouse. *p < 0.05; **p < 0.01. [file Image_1.docx]
